# The role of autophagy during murine primordial follicle assembly

**DOI:** 10.18632/aging.101376

**Published:** 2018-02-05

**Authors:** Yuan-Chao Sun, Yong-Yong Wang, Xiao-Feng Sun, Shun-Feng Cheng, Lan Li, Yong Zhao, Wei Shen, Hong Chen

**Affiliations:** 1 College of Animal Science and Technology, Northwest A&F University, Shaanxi Key Laboratory of Molecular Biology for Agriculture, Yangling Shaanxi, China; 2 Institute of Reproductive Sciences, College of Life Sciences, Qingdao Agricultural University, Qingdao, China; 3 Department of Reproductive Medicine, Qingdao Municipal Hospital, School of Medicine, Qingdao University, Qingdao, China

**Keywords:** primordial follicle, germ cell cyst breakdown, autophagy, apoptosis, epigenetic regulation

## Abstract

It is generally accepted that significant germ cell loss occurs during the establishment of the primordial follicle pool in most mammalian ovaries around the time of birth. However, the underlying mechanisms responsible for these processes remain largely unknown. In this investigation, we explored the role of autophagy during the establishment of the primordial follicle pool and found that autophagy was active in this process. Our data suggested that 17.5 dpc ovaries treated with rapamycin displayed a delay in germ cell cyst breakdown resulting in more oocytes at day 5 of treatment, while, ovaries that treated with 3-MA showed the opposite effect. We found that rapamycin treatment promoted autophagy and depressed cell apoptosis increasing the number of NOBOX positive oocytes. Furthermore, our results also revealed that epigenetic regulator, *Sirt1*, plays a role in germ cell loss. An epigenetic inhibitor or RNAi treatment of *Sirt1*, showed an increased level of H4K16ac and a decreased level of autophagy. Thus, these data indicate that autophagy prevents germ cell over loss during the establishment of primordial follicle pool, and this process may be influenced by *Sirt1*-invovled epigenetic regulation.

## Introduction

It is well known that about half to two-thirds of oocytes are lost during formation of the follicle pool in most mammalian ovaries, leading to a limited number of oocytes [1–8]. For example, in mice, the germ cell number was reported to decrease from 6070 ± 1120 to 1730 ± 550 per ovary from 13.5 days post coitum (dpc) to 3 days post partum (dpp) [4]. Whereas in humans a similar situation has been reported that there are about 6 - 7 million germ cells at week 20 of gestation, while only one-third of them remains at birth [9]. Many factors and signaling have been shown to be involved in the process of establishing the primordial follicle pool [10–18]. In *Drosophila*, it has been found that one oogonia can form a 16-cell-cyst through mitotic divisions, whereas, only one of these cells survives to form an oocyte, and the other cells inside the cyst die and provide necessary nutrients to the surviving oocyte [19]. In mice, it has been demonstrated that in a cyst, the germ cells are connected with each other via intercellular bridges, which can be observed in the early stages of oocyte development in mouse ovaries [20]. Similarly, only few germ cells within a cyst can survive for a couple of days following birth. A number of reports have demonstrated that the surviving oocytes have the common characteristic of containing Balbiani bodies, which are aggregates of organelles, such as mitochondria, endoplasmic reticulum (ER), and granulofibrillar material (GFM) [21–23]. In mice, the surviving early germ cells that possess the Balbiani have been demonstrated to assimilate nutrients from the other germ cells within a cyst [24].

One of the most important transitions is the change from the fetal ovarian environment to neonatal namely the transition from a nutrition supply from the maternal-fetal blood interface to lactation [25]. This transformation results in a limited supply of nutrients and resources which may be an important aspect of oocyte loss in the ovary. Programmed cell death (PCD), especially apoptosis, is reported to be responsible for the loss of oocytes [26–29], which is regulated by the balance of the expression of apoptotic genes, such as *Bax* and *Bcl-2* [30–32]. Autophagy, an important process, is crucial for the degradation of organelles [33–35], and has been reported to function in the development of germ cells [36–44]. A previous study revealed that female *Atg7* (autophagy related gene 7) knock-out mice are infertile. And germ cells with mutated *Atg7* showed a failure in the formation of autophagosomes which suggested a connection between germ cell development and autophagy [44]. Several reports have proved that autophagy plays a vital role during germ cell loss [36,41,42]. Another report demonstrated that *Beclin1* knocked-out mouse ovaries showed about a 50% - 60% reduction in the number of oocytes at 1 dpp. Moreover, the mouse ovaries with mutated *Atg7* lacked discernable oocytes and follicles at 1 dpp [36]. *Atg7* knockout mouse ovaries showed a great reduction of LC3 expression and displayed a remarkably decreased number of oocytes at 17.5 dpc [41]. All of these reports demonstrated that inhibition of autophagy would cause germ cell loss during the germ cell cyst breakdown period, suggesting autophagy may act as a protection or survival mechanism during germ cell cyst breakdown and the formation of the primordial follicle pool in mammalian ovaries.

Furthermore, epigenetic regulation was reported to play important roles in controlling the activity of autophagy [45–47]. For example, SIRT1, a NAD-dependent deacetylase of histone H4 lysine 16 acetylation (H4K16ac), is reported to be sufficient to promote basal rates of autophagy. Mouse embryonic fibroblasts cells with a *Sirt1* mutation showed a failure in activating autophagy [46]. The induction of autophagy is linked to the reduction of H4K16ac, and histone acetyltransferase hMOF (MYST1) and *Sirt1* work together to regulate the outcome of autophagy, as well as the survival of cancer cells cultured *in vitro* [47]. In this study, we investigated the roles of autophagy during germ cell cyst breakdown and primordial follicle assembly, and the level of autophagy influenced by epigenetic regulation during the establishment of the primordial follicle pool.

## RESULTS

### Germ cell loss and existence of autophagy during primordial follicle assembly

**Figure 1:**
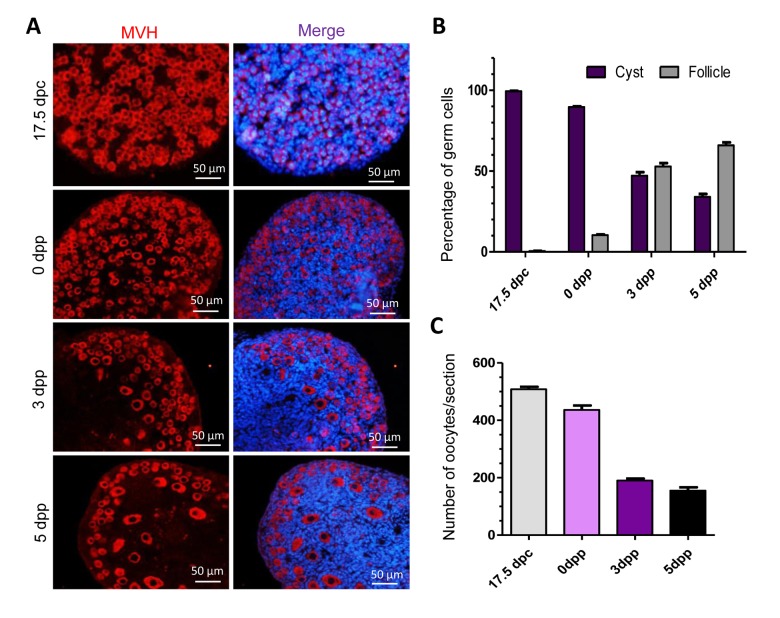
**Percentage and number of germ cells in different stages of mouse ovaries.** (**A**) Identification of germ cells with MVH in tissue sections of ovaries at 17.5 dpc, 0 dpp, 3 dpp and 5 dpp. (**B**) Percent of germ cells in cysts or in follicles at 17.5 dpc, 0 dpp, 3 dpp and 5 dpp; (**C**) Average of germ cell number per section in mouse ovaries at 17.5 dpc, 0 dpp, 3 dpp and 5 dpp.

Previous studies have demonstrated the important time frames of germ cell loss in mice. In this investigation, we investigated the dynamics of mouse germ cells in cysts and follicles at 17.5 dpc, 0 dpp, 3 dpp and 5 dpp. The germ cells were labeled with the germ cell specific mouse Vasa homolog (MVH) in cysts and follicles and were counted (Fig. 1A). In 17.5 dpc mouse ovaries, almost all the germ cells were held in germ cell cysts, in 0 dpp ovaries about 10% of the germ cells were within follicles, while in 3 dpp and 5 dpp ovaries about 50% and 70% of the germ cells were in follicles (Fig. 1B). In general, the breakdown of germ cell cysts and primordial follicle formation occur during the period of perinatal. We also counted the average number of total germ cells at these four-time points and found that remarkable germ cell loss happened during this period, especially at 3 dpp, showing a more than 50% reduction in germ cell numbers compared to 0 dpp ovaries (Fig. 1C).

**Figure 2:**
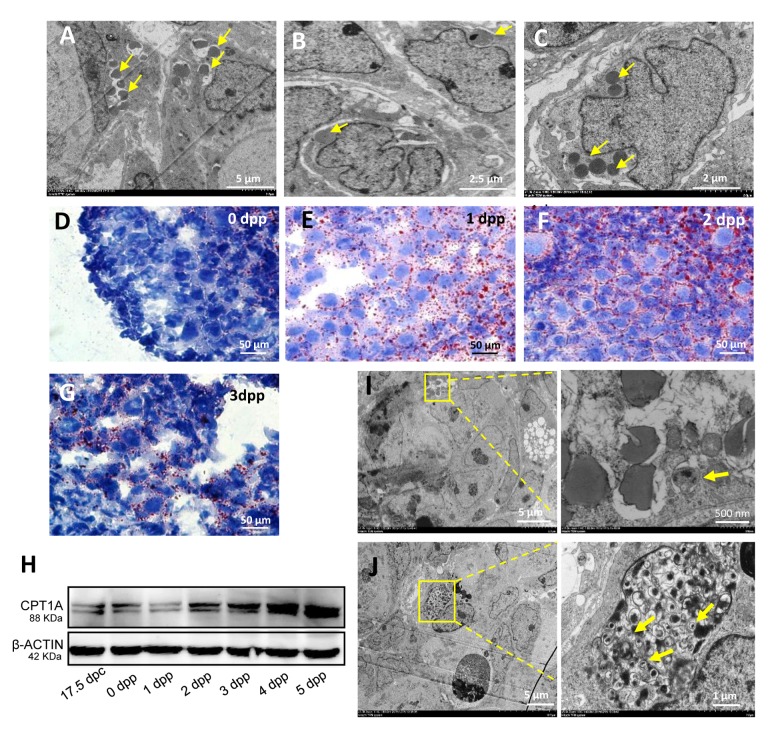
**Characteristics of germ cell loss in mouse ovaries.** (**A**) Observation of nuclear lipidation by TEM. (**B**) Observation of nuclear lipidation by TEM. (**C**) Observation of nuclear lipidation by TEM. (**D**-**G**) Oil red O staining of 0 dpp, 1 dpp, 2 dpp and 3 dpp, respectively. (**H**) WB detection for CPT1A in 17.5 dpc and 0 - 5 dpp ovaries. (**I**) Observation of nuclear lipidation with autophagosomes (frame) by TEM. (**J**) Observation of apoptosis with autophagosomes (frame) by TEM.

TEM was used to detect the 1 dpp and 2 dpp mouse ovaries, and it was found that during this period of intense germ cell loss a large number of germ cells within the cysts showed the phenomenon of nuclear lipidation (Fig. 2A - 2C). These dying germ cell nuclei were broken into fragments and formed lipid droplets. Oil red O staining confirmed that a high level of lipid droplets in 1 dpp and 2 dpp ovaries (Fig. 2D - 2G). We also detected that the regulator of fatty acid oxidation, carnitine palmitoyltransferase 1A (CPT1A), was highly expressed in the 17.5 dpc to 4 dpp mouse ovaries (Fig. 2H). Moreover, we found the existence of autophagosomes (Fig. 2I) which were also found in the germ cells undergoing apoptotic cell death (Fig. 2J).

**Figure 3:**
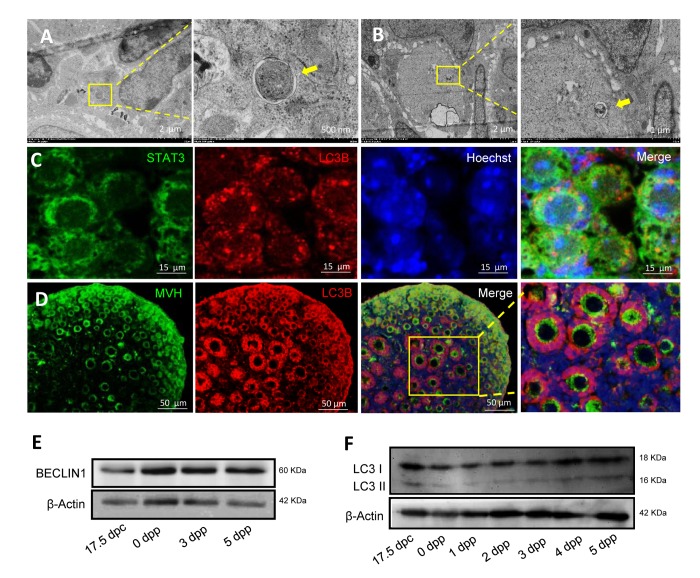
**Morphological characteristics and expression of autophagic markers in mouse ovaries.** (**A**) Observation of typical autophagosomes in 1 dpp mouse ovary by TEM. (**B**) Observation of typical autolysosome in 3 dpp mouse ovary by TEM. (**C**) Double IF staining for LC3B (red) and STAT3 (green) in 1 dpp mouse ovaries. (**D**) Double IF staining for LC3B (red) and MVH (green) in 3 dpp mouse ovaries. (**E**) WB detection for BECLIN1 in 17.5 dpc, 0 dpp, 3 dpp and 5 dpp mouse ovaries. (**F**) WB detection for BECLIN in 17.5 dpc, 0 - 5 dpp mouse ovaries.

**Figure 4:**
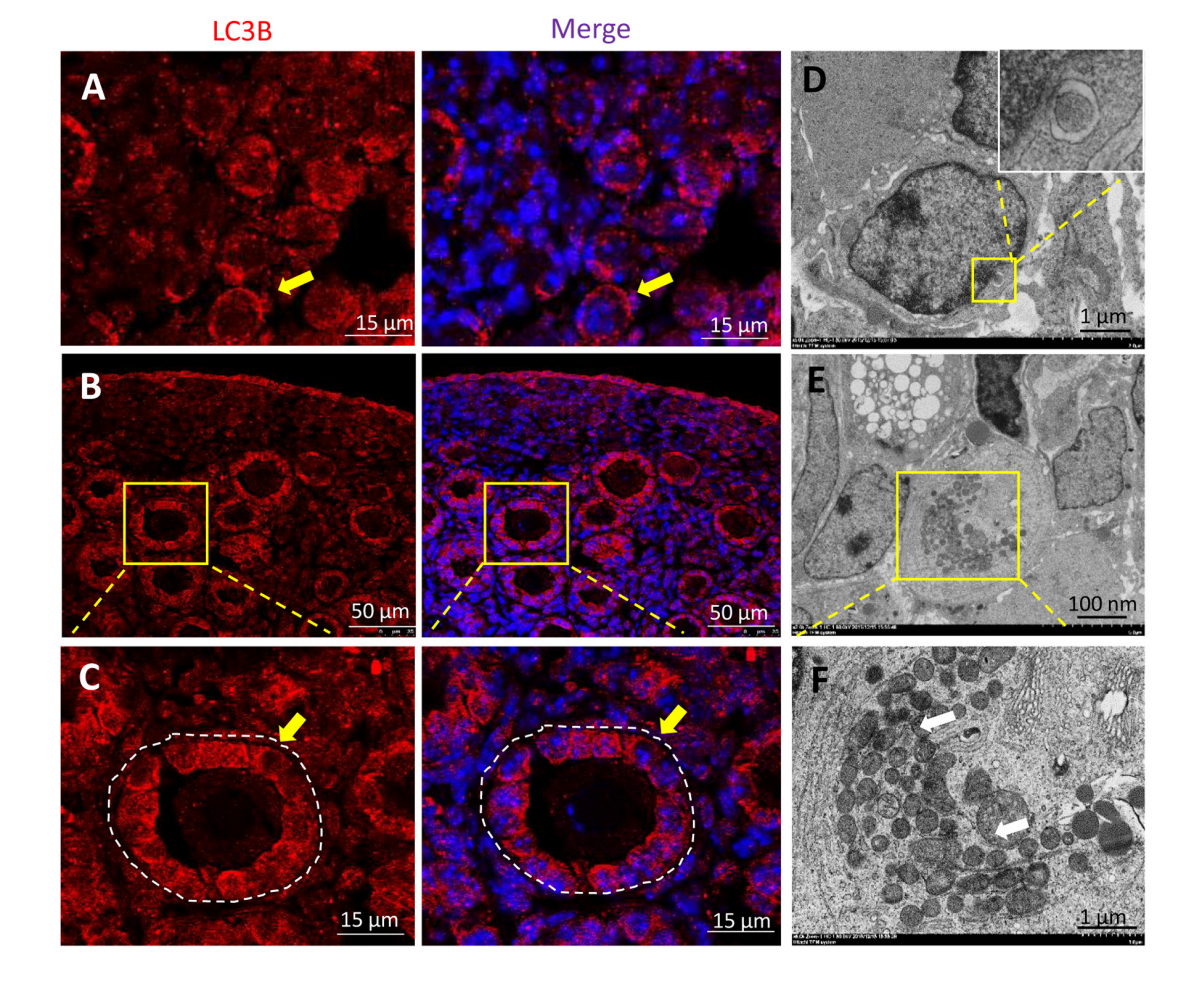
**Transformation of LC3B expression in 1 and 3 dpp ovary.** (**A**) IF staining for LC3B (red) in 1 dpp mouse ovaries. (**B**) IF staining for LC3B (red) in 3 dpp mouse ovaries. (**C**) IF staining for LC3B (red) in 3 dpp mouse ovaries. (**D**) Observation of typical autophagosomes in a granular cell within a follicle by TEM. (**E**-**F**) Observation of plenty mitochondria in an oocyte within follicle by TEM.

Autophagosomes were found to be frequently present in 1 dpp and 3 dpp mouse ovaries, with double layer membranes and cellular components inside (Fig. 3A and 3B). Double IF staining of light chain 3 protein B (LC3B) and STAT3 of 1 dpp mouse ovaries showed that LC3B and STAT3 were both located in the cytoplasm of oocytes within cysts (Fig. 3C). Double IF staining of LC3B and MVH of 3 dpp mouse ovaries showed that LC3B was located in the cytoplasm of oocytes within cysts, granulosa cells (but not oocytes) of both primordial and primary follicles, whereas MVH was expressed in these oocytes of primordial and primary follicles (Fig. 3D). ATG6, also known to be BECLIN1, was also expressed during the period of perinatal (Fig. 3E and 3F). Interestingly, there was a transformation of LC3B from oocytes within cysts to granulosa cells of new follicles, especially in 1 dpp and 3 dpp mouse ovaries (Fig. 4A - 4C). This situation was associated with the nutrient transport of surviving oocytes, supporting oocytes within cysts getting nutrients from neighboring oocytes, but oocytes in follicles getting nutrients from the granulosa cells around them. In Fig. 4D, in the primary follicles the granulosa cells showed autophagy to degrade cellular elements, and the surviving oocytes received nutrients containing plenty of mitochondria to supply energy for their development (Fig. 4E and 4F). From these results suggest that autophagy acts in the formation of primordial follicles and may be important for nutrient supply.

### Function of autophagy in the primordial follicle assembly

**Figure 5:**
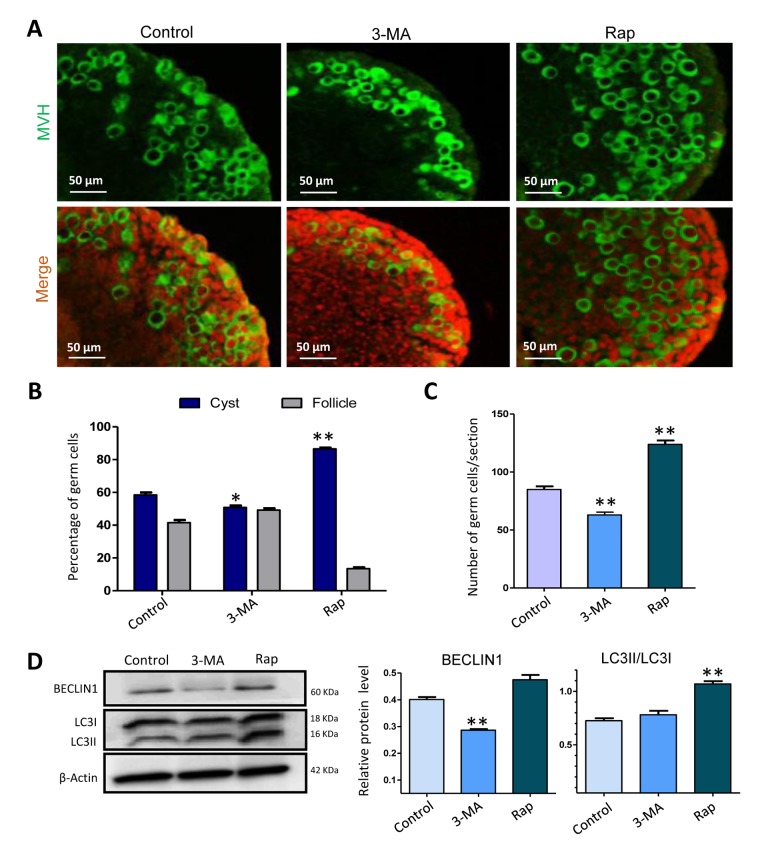
**Autophagy depressed germ cell cyst breakdown and increased the number of surviving gem cells after 3 days of treatment.** (**A**) IF staining for MVH (green) of control, rapamycin and 3-MA treated mouse ovaries for 3 days. (**B**) Percentage of germ cells in cysts and follicles in the three groups after 3 days treatment. (**C**) Average number of survived oocytes in the three groups. (**D**) Level of BECLIN1 protein in control, rapamycin and 3-MA treated ovaries (6 h). The results are presented as mean ± SD. *P < 0.05; **P < 0.01.

To investigate the influence of autophagy during the formation of primordial follicles, we cultured 17.5 dpc fetal mouse ovaries for two days *in vitro*, and then continually cultured them for an additional 3 or 5 days in addition of rapamycin or 3-MA. It was interesting that after 3 days of culture with rapamycin the ovaries showed a delay of germ cell cyst breakdown compared to that of the control group, whereas ovaries treated with 3-MA showed the opposite result, where germ cell cyst breakdown was promoted (Fig. 5A and 5B). The average number of total germ cells in rapamycin treated ovaries was more than that of the control group, while the average number of total germ cells in 3-MA treated group was less than that of the control group and rapamycin treatment group (Fig. 5C). Then we investigated the expression level of autophagy related proteins and found that compared to the control group, ovaries treated with rapamycin for 6 h displayed a higher level of BECLIN1 and LC3-II/LC3-I, suggesting an increased level of autophagy. Ovaries treated with 3-MA showed lower level of BECLIN1, but LC3-II/LC3-I was not altered (Fig. 5D).

**Figure 6:**
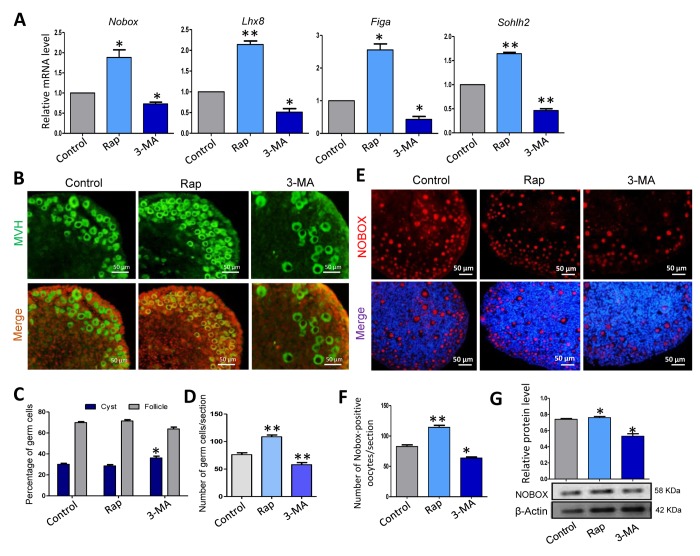
**Rapamycin-promoted autophagy and prevented germ cell over loss after 5 days of treatment.** (**A**) Quantitative RT-PCR for *Nobox*, *Lhx8*, *Figa* and *Sohlh2* mRNA levels in control, rapamycin and 3-MA treated ovaries for 5 days. (**B**) IF staining for MVH (green) of control, rapamycin and 3-MA treated mouse ovaries for 5 days. (**C**) Percentage of germ cells in cysts and follicles in the three groups after 5 days treatment. (**D**) Average number of survived oocytes in the three groups. Autophagy leads to more survival of gem cells. (**E**-**F**) Number of NOBOX-positive oocytes/section in control, rapamycin and 3-MA treated ovaries. (**G**) Level of NOBOX protein in control, rapamycin and 3-MA treated ovaries. The results are presented as mean ± SD. * P < 0.05; ** P < 0.01.

Although there was no trend in the expression of follicle formation relevant genes after 3 days of culture (data not show), rapamycin treated ovaries after 5 days culture showed a much higher level of expression of *Nobox*, *Lhx8*, *Figlα* and *Sohlh2*, whereas the 3-MA treated group had a decreased expression level of these genes compared to that of the control group (Fig. 6A). We also investigated the status of primordial follicle formation in the three groups (Fig. 6B), and rapamycin treated group displayed a very similar ratio of oocytes in follicles after 5 days culture compared to that of the control group, but with a much larger number of total oocytes surviving, while the 3-MA treated group displayed fewer oocytes surviving (Fig. 6C and 6D). Furthermore, rapamycin treated ovaries for 3 days had a higher number of NOBOX positive cells and higher expression of NOBOX, while the 3-MA treated group showed the opposite result (Fig. 6E - 6G).

**Figure 7:**
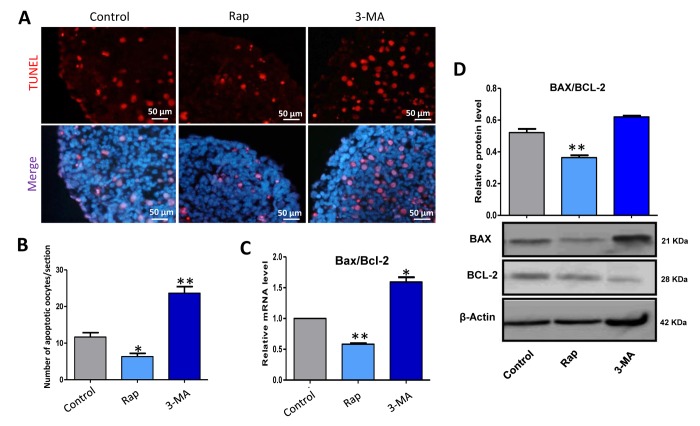
**Rapamycin-promoted autophagy decreased the level of apoptosis.** (**A**) TUNEL histochemistry (red) in tissue sections of ovaries from control, rapamycin and 3-MA treated ovaries for 3 days. (**B**) Autophagy decreased the number of TUNEL positive cells. (**C**) Quantitative RT-PCR for *Bax/Bcl-2* mRNA levels in control, rapamycin and 3-MA treated ovaries for 3 days. (**D**) WB analysis of BAX/BCL-2 in control, rapamycin and 3-MA treated ovaries for 3 days. The results are presented as mean ± SD. *P < 0.05; **P < 0.01.

TUNEL assay showed that rapamycin treated ovaries for 3 days showed a lower level of apoptosis compared to that of the control group (Fig. 7A and 7B), which was confirmed with the ratio of *Bax/Bcl-2* mRNA and protein (Fig. 7 and 7D). These data indicate that rapamycin induced autophagy to delay germ cell cyst breakdown, and resulted in more oocytes surviving.

### Role of epigenetic regulation during primordial follicle assembly through autophagy

**Figure 8:**
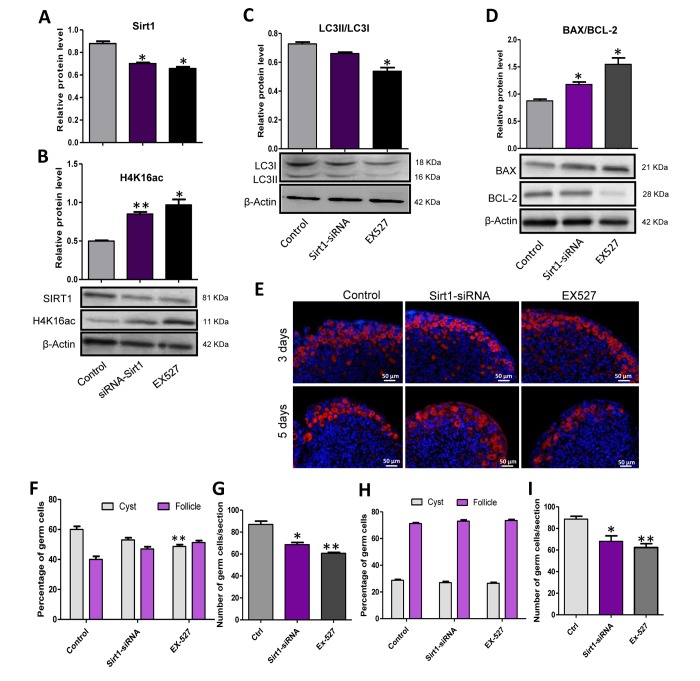
***Sirt1* inhibition depressed autophagy and caused an over loss of germ cells.** (**A**) Level of SIRT1 protein in control, EX527 and *Sirt1* RNAi treatment for 2 days ovaries. (**B**) Level of H4K16ac protein in control, EX527 and *Sirt1* RNAi treatment for 2 days ovaries. (**C**) WB analysis of LC3II/LC3I in control, EX527 and *Sirt1* RNAi treated ovaries (6 h). (**D**) WB analysis of BAX/BCL-2 in control, EX527 and *Sirt1* RNAi treated ovaries for 3 days. (**E**) IF staining for MVH (red) of control, EX527 and *Sirt1* RNAi treated mouse ovaries for 3 days and 5 days. (**F**) Percentage of germ cells in cysts and follicles in the three groups after 3 days treatment. (**G**) Average number of survived oocytes in the three groups after 3 days treatment. (**H**) Percentage of germ cells in cysts and follicles in the three groups after 5 days treatment. (**I**) Average number of survived oocytes in the three groups after 5 days treatment. The results are presented as mean ± SD. * P < 0.05; ** P < 0.01.

*Sirt1* was reported to influence the level of autophagy and to work together with hMOFs in regulating the outcome of autophagy therefore we investigated the role of *Sirt1* in autophagy and the formation of primordial follicle. Firstly, 17.5 dpc mouse ovaries were cultured *in vitro* for two days, then *Sirt1* inhibitor, EX527 or *Sirt1* RNAi were added into the culture for an additional 3 or 5 days. The ovaries that were treated with EX527 or *Sirt1* RNAi showed a decreased level of SIRT1 protein (Fig. 8A) and an increased level of H4K16ac (Fig. 8B) compared to the control group after 2 days of treatment. We also detected the level of autophagy of these three groups and found that the ratio of LC3II/LC3I was decreased in the EX527 or *Sirt1* RNAi treated group (Fig. 8C), indicating a depression of autophagy by *Sirt1* inhibition. Moreover, EX527 or *Sirt1* RNAi decreased the ratio of BAX/BCL-2 (Fig. 8D). We also investigated the status of germ cell loss during the treatment (Fig. 8E) and found that after 3 days of culture, EX527 or *Sirt1* RNAi group had enhanced germ cell cyst breakdown and the average number of total germ cells in these two groups was decreased in comparison to the control group (Fig. 8F and 8G). After 5 days treatment, EX527 or *Sirt1* RNAi group had a very similar ratio of oocytes in cysts and follicles compared to that of control group, but an obvious reduction in the number of surviving oocytes (Fig. 8H and 8I).

## DISCUSSION

In this investigation, the observation of autophagosomes and the detection of autophagy relatively specific markers suggested a role for autophagy during the process of germ cell cyst breakdown and primordial follicle assembly. We found that 17.5 dpc ovaries that were treated with rapamycin displayed a high level of autophagy and a delay in germ cell cyst breakdown, but increased the surviving oocytes compared to that in the control group, and 3-MA treated group showed the opposite effects. Further investigation revealed that rapamycin treated group showed a depression of apoptosis and an increased number of NOBOX positive oocytes. We also found that epigenetic regulation, the level of H4K16ac influenced the level of autophagy as well as the number of surviving germ cells in cultured ovaries *in vitro*.

Several reports have demonstrated that remarkable germ cell loss occurs during the formation of the primordial follicle pool in mammalian ovaries around the time of birth [1–7]. The reduction of germ cells during this period can reach up to half or two third of the whole germ cell population [4,8]. In our investigation, 3 dpp mouse ovaries showed a reduction of 50% germ cells compared to 17.5 dpc ovaries. Histological observation showed that from 17.5 dpc to 3 dpp, the majority of germ cell cysts underwent breakdown to form primordial follicles, but not every germ cell within cysts could develop into an oocyte. We found that within 1 dpp and 2 dpp mouse ovaries, a great number of germ cells in the cysts displayed nuclear lipidation, which maybe one mechanism of initiating apoptosis. One report revealed that in rat ovaries, primordial follicles displayed a weak expression of LC3, whereas, granulosa cells in primary and preantral follicles exhibited very intense LC3 expression, the oocytes within these follicles showed a deficiency [29]. Similar results were found in our study, in 1 dpp mouse ovaries, LC3B showed positive expression in oocytes within cysts. But in primordial and primary follicles, LC3B was only positive in the granular cells, but not in the oocytes. This transform of LC3B expression inosculated with the transform of surviving oocytes nutrients supply, saying oocytes within cysts received nutrients from neighbor cyst cells or ‘eating’ themselves, but oocytes in follicles got nutrients from the granular cells around them.

Autophagy was reported to play a vital role in the formation of primordial follicles, and to act in protecting germ cells from over loss [25,30,31]. In our investigation, rapamycin promoted autophagy showing a delay in germ cell cyst breakdown after 3 days of treatment, and more oocytes survived after 3 and 5 days treatment, whereas the 3-MA group showed the opposite result. Further investigation demonstrated that rapamycin treated mouse ovaries showed a higher level of autophagy and a decreased level of apoptosis. These data indicated that rapamycin promoted autophagy and repressed apoptosis and delayed the germ cell cyst breakdown, keeping more oocytes surviving during the formation of primordial follicles. Several reports demonstrated that epigenetic regulation influences the level of autophagy [33–35]. For example, *Sirt1* and hMOF work together to regulate the level of H4K16ac and to control the outcome of autophagy [35]. In addition, a number of reports demonstrated that *Sirt1* directly regulates autophagy through non-epigenetic way, for example, treatment with the AMPK activator aminoimidazole carboxamide ribonucleotide (AICAR) or glucose starvation in culture raised the effect of GAPDH association on *Sirt1* activity and showed a decrease in LC3 acetylation, but not histone acetylation to regulate the level of autophagy [48,49]. Another report demonstrated that *Sirt1* depletion mice showed a deacetylation of LC3 and Atg7, as well as a failure in both spermatogenesis and activation of autophagy [50]. Furthermore, histone modification, such as H3K4me3 was reported to directly determine the survival of the arrested oocytes [51]. In our investigation, we used the inhibitor of *Sirt1*, EX527, as well as RNAi to explore the role of epigenetic regulation in autophagy and primordial follicle formation, and found that, depressing *Sirt1* expression showed a decreased level of H4K16ac, also a decreased level of autophagy and less surviving oocytes. These data indicated a connection between *Sirt1* suppression and development of primordial follicles through autophagy. However, further work is still needed to investigate whether *Sirt1* regulates the size of primordial follicle pool through epigenetic or non-epigenetic way.

**Figure 9:**
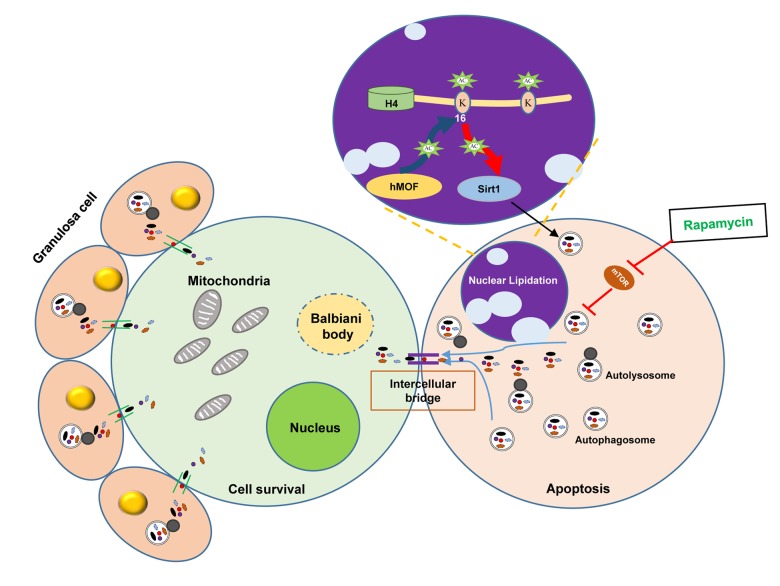
Scheme illustrating the role of autophagy in protecting oocytes over loss during germ cell cyst breakdown and primordial follicle formation.

In conclusion, the data in this investigation demonstrate that autophagy plays a part in germ cell cyst breakdown and primordial follicle assembly. Rapamycin promoted autophagy with a delay in germ cell cyst breakdown and an increased number of surviving oocytes, which might be regulated by *Sirt1* mediated epigenetic regulation. The transformation of LC3B from oocytes within cysts to granular cells of follicles may be associated with the nutrient transport of surviving oocytes, suggesting autophagy promotes the cyclic utilization of nutrient, so as to depress unnecessary apoptosis, avoiding germ cell over loss during germ cell cyst breakdown and establishment of the primordial follicle pool (Fig. 9).

## MATERIALS AND METHODS

### Animals

CD1 mice (Vital River Laboratory Animal Technology Co. Ltd, Beijing, China) were housed at Qingdao Agriculture University, under conditions of controlled temperature (21 - 22 °C) and light (half a day for light, half a day for dark, cycle). Only female mice that mated with male mice and displayed a vaginal plug the next morning were used, and were considered 0.5 dpc. All operations involving animal experiments were within the specification of Institutional Animal Care and Use Committees of Qingdao Agricultural University.

### Ovary isolation and culture *in vitro*

Briefly, 17.5 dpc mouse ovaries were dissected and cultured in 24-well plate (Boyang, BYA3, China), at 37 °C with 5% CO_2_. Culture medium is decided according to our previous report [17]. After 2 days of culture, the cultured ovaries were treated with rapamycin (Gene Operation, IPA1021-0010MG, USA) at 300 nM, 3-MA (Gene Operation, IPA1009-0050MG, USA) at 10 mM, EX527 (Tocris, 2780, UK) at 10 μM, and adding 0.1% DMSO as negative control, respectively. With the *Sirt1* siRNA treatment, RNAi for *Sirt1* (#1: 5’-GCACCGAUCCUCGAACAAUTT-3’; #2: 5’-AUUGUUCGAGGAUCGGUGCTT-3’) was mixed with Lipofectamine 2000 (Invitrogen, 11668-027, USA) and added into the culture medium at a concentration of 200 pmol / 500 ml. RNAi for *Sirt1* and for a negative control were designed and supplied by Genepharma (Shanghai, China).

### Immunofluorescence (IF)

Ovaries were cleaned and washed with PBS 3 times, then fixed in 4% paraformaldehyde at 4 °C overnight. The ovaries were treated with histological procedures, paraffin embedding and were sectioned into 5 μm sections. These sections were held at 60 °C for 30 min, and placed in xylene and rehydrated. Then, sections were transferred into sodium citrate buffer at 96 °C for 10 min. Following 45 min of blocking with BDT (TBS with 10% goat serum and 3% BSA), sections were incubated with primary antibodies (Table S2) at 4 °C overnight. Then sections were incubated with Cy3-labeled or FITC-labeled secondary antibodies (dilution 1: 150, Beyotime, A0516, China) for half an hour at 37 °C. Counterstaining was treated with Hoechst33342 (Beyotime) or PI (Abcam, ab14083, USA) for 5 min.

### Transmission Electron Microscopy (TEM)

The ovaries were dissected and washed three times in PBS, and fixed in 2.5% glutaraldehyde (within 0.2 M PBS, pH = 7.2) overnight at 4 °C. Then the treated ovaries were processed and wrapped in epoxypropane resin following standard TEM procedures. The serial sections were cut at 50 nm using the EM UC7 ultramicrotome (Leica, Germany), stained with lead citrate and uranium and observed under HT7700 TEM (Hitachi, Japan).

### TUNEL staining

Bright Red Apoptosis Detect Kit (Vazyme, A113-02, China) was used for the detection of TUNEL positive cells. Briefly, sections of treated ovaries were heated at the 60 °C for 30 min, then placed in xylene and rehydration through a demotion series of ethanol, and wash with PBS for 10 min. Samples were treated by proteinase K at room temperature for a quarter and washed twice with PBS. Then carried on an incubation with the TUNEL treatment mixture at 37 °C for an hour. Counterstaining was treated with Hoechst33342.

### Western blot

Using the Cell Lysis Buffer for Western (Beyotime, P0013, China) to obtain protein extracts from 4 ovaries. Then samples were carried on 10% SDS-PAGE gel and transferred onto Immobilon-PSQ Transfer Membrane (Millipore, USA). After blocking, these obtained membranes were carried on an incubation with primary antibody (Table S2) at 4 °C overnight. Then, the membranes were collected and washed in TBST (Tris-Buffered Saline and Tween 20), following an incubation with horseradish peroxidase (HRP)-conjugated goat anti-rabbit IgG (Beyotime, A0216, China) or goat anti-mouse IgG (Beyotime, A0258, China) at 1: 2000 dilution in TBST, at 37 °C for 2 h. Then, BeyoECL Plus Kit (Beyotime, P0018, China) was used for the exposure. β-ACTIN were used as housekeeping protein control.

### RNA extraction and quantitative real-time PCR

The primers used in this research are listed in Table S1. Briefly, RNA Prep Pure Micro Kit (Aidlab, RN07, China) was used to extract mRNA from two ovaries, and then these mRNA were reverse-transcribed into cDNA using TransScript One-Step gDNA Removal and cDNA Synthesis SuperMix (TransGen, AT311-03, China) according to the manufacturer’s descriptions. The PCR conditions were set according to the description and previous report [17]. Quantitative PCR was operated with Light Cycler real-time PCR instrument (Roche, LC480, Switzerland) using the Light Cycler SYBR Green I Master (Roche, Switzerland). Gene expression changes were analyzed by the 2^-△△Ct^ method and normalized to *β-Actin.*

### Statistical analyses

Data are represented as mean ± SD. T-test was used to assess the difference between two groups (normal distribution) and one-way analysis of variance (ANOVA) for multiple comparison tests. Statistical analysis of follicle number counts was performed using Prism 5.0. Comparisons were considered significant at *P* < 0.05.

## Supplementary Material

Supplementary File
