# Correction for: The role of autophagy during murine primordial follicle assembly

**DOI:** 10.18632/aging.206051

**Published:** 2024-08-15

**Authors:** Yuan-Chao Sun, Yong-Yong Wang, Xiao-Feng Sun, Shun-Feng Cheng, Lan Li, Yong Zhao, Wei Shen, Hong Chen

**Affiliations:** 1 College of Animal Science and Technology, Northwest A&F University, Shaanxi Key Laboratory of Molecular Biology for Agriculture, Yangling, Shaanxi, China; 2 Institute of Reproductive Sciences, College of Life Sciences, Qingdao Agricultural University, Qingdao, China; 3 Department of Reproductive Medicine, Qingdao Municipal Hospital, School of Medicine, Qingdao University, Qingdao, China

**Keywords:** primordial follicle, germ cell cyst breakdown, autophagy, apoptosis, epigenetic regulation

**This article has been corrected:** It was found that there was overlap between two images of MVH-stained mouse ovaries in **Figure 8E**, despite the ovaries being treated differently for 3 days. The authors confirmed that they had inadvertently misplaced the fluorescence image for the Control group (3 days of treatment). The authors provided original images for the Control and Sirt1-siRNA groups and confirmed that other results were not affected by this mistake. They replaced incorrect Control group (3 days of treatment) image in **Figure 8E** with the correct image of immunofluorescent MVH staining from the original experiments. The authors state that this correction has no impact on the experimental outcome or conclusions and would like to apologize for any inconvenience caused.

The corrected version of **Figure 8** is provided below.

**Figure 8:**
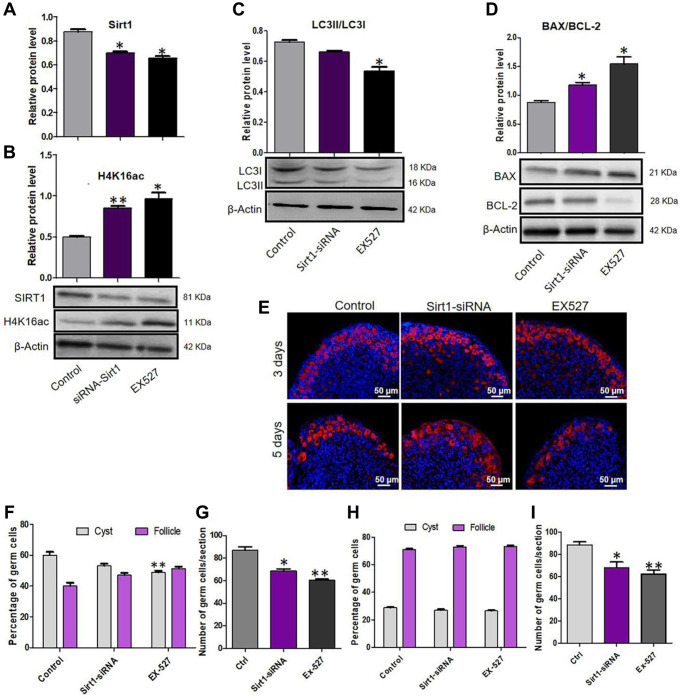
**Sirt1 inhibition depressed autophagy and caused an over loss of germ cells.** (**A**) Level of SIRT1 protein in control, EX527 and Sirt1 RNAi treatment for 2 days ovaries. (**B**) Level of H4K16ac protein in control, EX527 and Sirt1 RNAi treatment for 2 days ovaries. (**C**) WB analysis of LC3II/LC3I in control, EX527 and Sirt1 RNAi treated ovaries (6 h). (**D**) WB analysis of BAX/BCL-2 in control, EX527 and Sirt1 RNAi treated ovaries for 3 days. (**E**) IF staining for MVH (red) of control, EX527 and Sirt1 RNAi treated mouse ovaries for 3 days and 5 days. (**F**) Percentage of germ cells in cysts and follicles in the three groups after 3 days treatment. (**G**) Average number of survived oocytes in the three groups after 3 days treatment. (**H**) Percentage of germ cells in cysts and follicles in the three groups after 5 days treatment. (**I**) Average number of survived oocytes in the three groups after 5 days treatment. The results are presented as mean ± SD. ^*^P < 0.05; ^**^P < 0.01.

