# Supplementary material for: The role of autophagy during murine primordial follicle assembly
Source: Aging (Albany NY). 2018 Feb 5;10(2):197–211. doi: 10.18632/aging.101376 (PMC5842841; doi:10.18632/aging.101376)
Supplement: Supplementary File [file aging-10-101376-s001.pdf]

## SUPPLEMENTARY MATERIAL

**Table S1. Primers used for quantitative RT-PCR.**

| Gene | Genbank | Forward primer sequence | Reverse primer sequence | Product Length(bp) |
|---|---|---|---|---|
| *Nobox* | NM_130869.3 | CTATCCTGACAGTGACAAACGCC | CACCCTCTCAGCACCCTCATTAT | 251 |
| *Lhx8* | NC_000069.5 | CAGTTCGCTCAGGACAACAA | CCTGCAGTTCTGAAACCACA | 105 |
| *Sohlh2* | NM_028937.3 | TCTCAGCCACATCACAGAGG | GGGGACGCGAGTCTTATACA | 199 |
| *Figla* | NM_012013.1 | ACAGAGCAGGAAGCCCAGTA | TGGGTAGCATTTCCCAAGAG | 225 |
| *Bax* | NM_007527 | ATGCGTCCAAGGAAGACTGAG | CCCCAGTTGAAGTTGCCATCAG | 162 |
| *Bcl-2* | NM_009741 | GCAGAGATGTCCAGTCAG | CACCGAACTCAAAGAAGG | 127 |
| *Actin* | NM_007393.3 | TCGTGGGCCGCTCTAGGCAC | TGGCCTTAGGGTTCAGGGGGG | 255 |

**Table S2. Antibodies.**

| Antibody | Vendor | Dilution |
|---|---|---|
| MVH(IHC) | Abcam (ab13840) | 1:200 |
| MVH(Double IF) | mAbcam (ab27591) | 1:200 |
| LC3B (WB) | Sigma (102M4778V) | 1:1000 |
| LC3B (IHC) | Abcam (ab51520) | 1:1000 |
| NOBOX (IHC and WB) | Abcam (ab41521) | 1:1000(WB)/1:200(IHC) |
| BAX (WB) | Cell Signaling (#2772S) | 1:1000 |
| BCL-2 (WB) | Immuno Way (YT0470) | 1:1000 |
| BECLIN1 (WB) | Cell Signaling (#3738) | 1:1000 |
| β-ACTIN (WB) | Boster (BM0627) | 1:1000 |
| CPT1A | Proteintech (15184-1-AP) | 1:1000 |
